# A fast and safe technique for sperm preparation in ICSI treatments within a randomized controlled trial (RCT)

**DOI:** 10.1186/s12958-020-00642-8

**Published:** 2020-08-19

**Authors:** Domenico Baldini, Annamaria Baldini, Erica Silvestris, Giovanni Vizziello, Daniele Ferri, Damiano Vizziello

**Affiliations:** 1 Momò Fertilife Clinic, Bisceglie, Italy; 2 Gynecologic Oncology Unit, IRCCS Istituto Tumori “Giovanni Paolo II”, Bari, Italy; 3 University of Milano, Milan, Italy

**Keywords:** Horizontal sperm migration, ICSI, Reproductive outcomes, Sperm preparation

## Abstract

Recently a novel method based on horizontal sperm migration in injection dishes has been introduced as an additional tool for preparation of semen sample in assisted reproductive technology (ART) procedures. In the present study, we evaluated both timing and reproductive outcomes in a randomized controlled study including 1034 intra-cytoplasmic sperm injection (ICSI) procedures followed by fresh embryo transfer. Couples enrolled were divided into two sub-groups, namely conventional swim-up method (Group A), and horizontal sperm migration in injection dishes (Group B).

No significant differences were found between groups with respect to fertilization rate, implantation success, clinical pregnancy outcomes and ongoing pregnancies. On the contrary, both cleavage and blastocyst rates were statistically higher in Group B, suggesting superior efficiency and safety of this innovative technique also including time-saving and cheaper costs as compared to the classical swim-up sperm preparation.

Our data support the interpretation of the horizontal sperm migration as a promising procedure for semen preparation in ART cycles.

## Introduction

The human ejaculate is a combination of several constituents such as spermatozoa, epithelial and blood cells also including a mixture of immature and necrotic components involved in the synthesis of reactive oxygen species (ROS). The effects of ROS production with respect to DNA stability include both decondensation and fragmentation damage [1] resulting in an impaired fertilization potential of the semen ([2]; Aitken & Clarckson, 1987a [3, 4];). The damaging mechanism of ROS includes the lipid peroxidation of polyunsaturated fatty acids within the sperms’ plasmatic membrane with the final result of a reduction of the fast-rectilinear motility and altered morphology [5–7] which, however, are not apparently paralleled by potential damages of the sperm DNA [8–12].

Several Authors also reported how elevations of DNA fragmentation in sperm can induce adverse effects on assisted reproduction outcomes including lower fertilization rate and impaired embryo cleavage and implantation (IR) [13–15]. Among conventional techniques for sperm preparation in ART procedures and cycles, the swim-up technique, based on the active migration of spermatozoa from a pre-washed cell pellet within an overlaying medium in relation to the different spermatic gradient density [16], is currently considered a well-established and efficient method [17].

However, although this conventional technique is largely adopted, it also includes several drawbacks related to the increased cell-to-cell contact within the spermatozoa pellet leading to high ROS production [18] due to repeated cell centrifugations and long incubation time for samples. Moreover, other disputed aspects of intra-cytoplasmic sperm injection (ICSI) have been related to controversial outcomes [19, 20].

Therefore, considering that both timing of each procedure and safety of the biological samples in order to avoid the risk of samples mixture are critical for the in vitro fertilization (IVF) procedure, we performed an easier and faster approach for the semen management which avoids the centrifugation steps and the potential DNA damage induced by the cellular stress, that is based on the horizontal sperm migration directly in the injection dish [21].

Thus, our study is aimed at verifying the efficiency of this novel sperm preparation technique in comparison with the standard swim-up method in ICSI cycles.

## Materials and methods

### Determination of the ideal sample size

In our randomized controlled trial, the sample size was primarily assessed by a mathematical tip (Cochran’s sample size formula). Briefly, we first calculated the ideal sample size from the total number of patients undergoing ART procedures in the geographic area of interest (Southern Italy), which include about 3000 cycles per year. Then, by adopting a 95% confidence interval level and a 3% margin of error, we obtained 997 cycles as ideal sample, which is slightly lower than the total number of cycles included in this study. Therefore, by considering two distinct Groups A and B of respectively 498 and 536 couples, the allocation report in this study is 1:1.07.

**Fig. 1:**
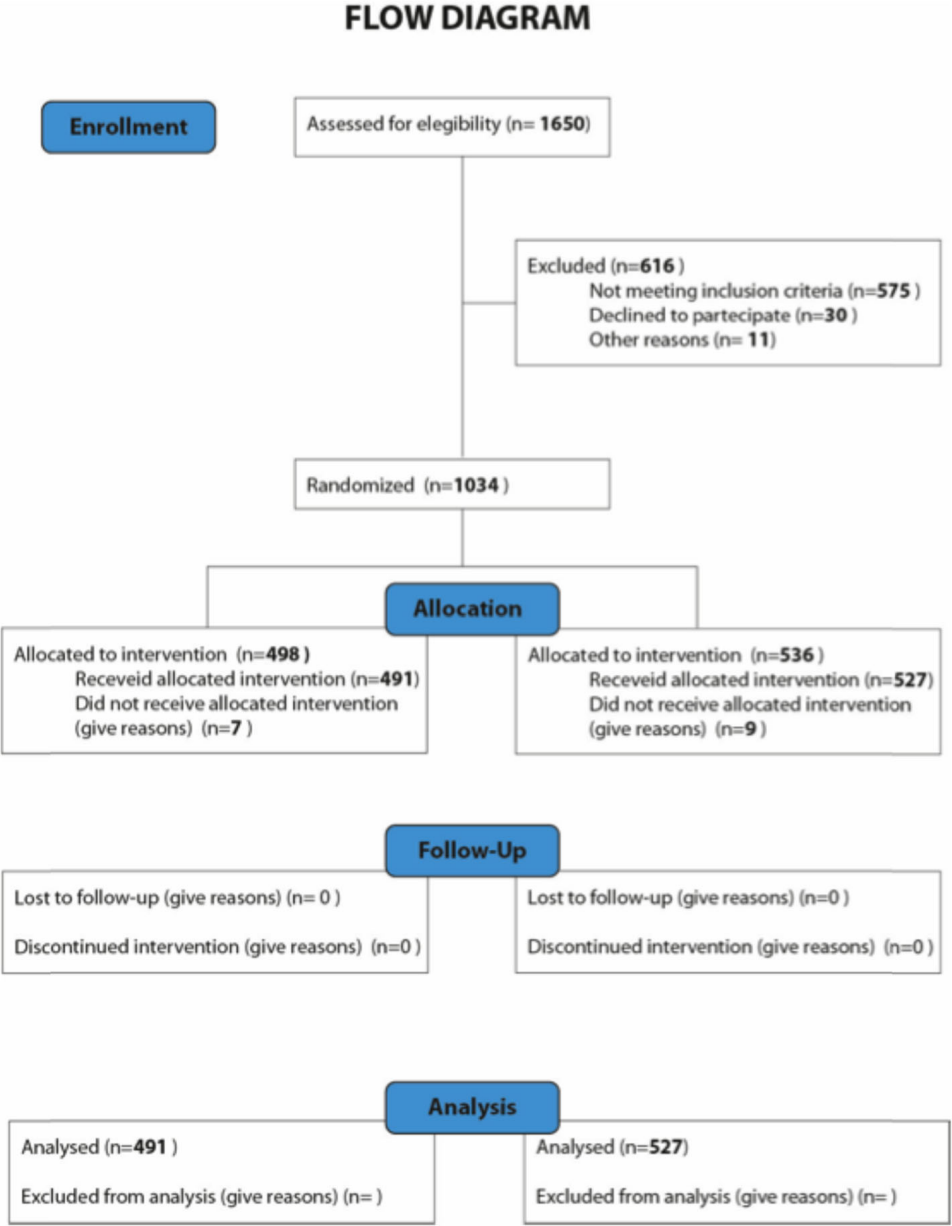
Flow diagram based on the determination of our sample size used in the study which was split into 2 different groups: A (498) and B (536)

Our study included patients enrolled at our reproductive centre (Momò Fertilife, Bisceglie, Italy), who were recruited from a large geographic area, mainly southern Italy. Since January 2015 to December 2019, we performed 2539 ART cycles including 1650 with fresh embryo transfers (ET), and 880 with frozen ETs. However, considering as inclusion criteria women < 38 yrs. (without previous ovarian surgery, endometriosis and/or a premature ovarian failure (POF)), and sperm concentration > 1 × 10 ^6^ million sperm/ml for men, the effective enrolled cycles were 1034, whereas 41 couples denied the consent to the study and additional 1464 couples failed to satisfy the inclusion criteria of the study (Fig. 1).

The couples were randomized into two groups according to the sperm preparation for ICSI. Group A (*n* = 498) included sperm managed by the conventional swim-up method, whereas Group B (*n* = 536) enrolled patients whose sperms were treated by the horizontal swim-up preparation procedure. The women’s age ranged between 24 and 38 years with a mean of 34.09 ± 3.08 years, whereas the male age ranged between 24 and 58 years (Table 1). Geographic derivation of patients was prevalently from Apulia region (Table 1). All patients provided their written consent to the study that was approved by the local Ethical Committe, and were anonymized in our database (MedITEX-IVF).

**Table 1:** Demographic and clinical characteristic of the sample

	A Group (***n*** = 498)	B Group (***n*** = 536)
**Female age (years)**	34.07 (±3.24)	34.10 (±2.93)
**Male age (years)**	36.79 (±4.52)	36.9 (±3.96)
**Geographical area of ​​origin**		
Region	83%	82%
Neighboring regions	10%	11%
Outside the region	7%	7%
**Aetiology female infertility**		
Tubal	9,9%	10,2%
Reduced ovarian reserve	25%	25,1%
PCOS	10%	9,8%
Various	10%	9,8%
None	50%	50%
**Aetiology male infertility**		
OA Severe	22,5%	21,4%
OA Moderate	17,2%	17,7%
Various	11%	10%
None	41,6%	43,1%
**Years of infertility**		
from 1 to 3	55,1%	53,8%
from 4 to 6	28,7%	29,1%
from 7 to 9	11,7%	11,8%
over 9	4,5%	5,2%

### Ovarian stimulation protocol and oocyte collection

Females from both groups were conventionally treated with the gonadotropin-releasing hormone (GnRH) antagonist (Cetrotide, Merck Serono) and stimulated with recombinant follicle stimulating hormone (FSH) preparation (GONAL-f, Merck Serono). Thus, oocytes were collected between 34 and 37 h after the human chorionic gonadotropin (hCG) administration and the oocyte retrieval was performed through a vaginal ovarian pick up (OPU) under ultrasound guidance (VOLUSON S8, GE Healthcare).

After 3 h from the oocyte retrieval, the cumulus-oocyte complexes were exposed to 25 IU/ml hyaluronidase solution (LifeGlobal Group) to remove the corona radiata by repeated pipetting. The retrieved oocytes were then inspected under a stereomicroscope (Nikon SMZ 1500) and only those in metaphase II (MII)-stage, namely mature eggs, were injected after 40 h from hCG administration.

### Semen preparation procedure

Semen samples from both groups were collected after a sexual abstinence of approximately 3–5 days in accordance with the World of Health Organization (WHO) 2010 guidelines. The samples were maintained at 37 °C for 20–30 min in order to complete the liquefaction process and then evaluated under a phase-contrast microscope (Nikon eclipse E 200), while and 10 μl of sperm suspension was recovered to assess the sperm count and motility to complete the preparation procedure.

Seminal fluids from Group A patients, were prepared using the conventional swim-up technique by placing samples in a conical tube and supplementation of the same volume of preparing medium (Global, LifeGlobal) followed by centrifuging at 300 g for 10 min. Supernatants were then discharged and a volume of 0.2–0.5 ml of preparing medium was added to the pellet. The tube was then placed on a stand and tilted at an angle of 45° and incubated for 30 min. Further, the supernatant was aspirated by a sterile pipette and transferred into a sterile conic tube.

**Fig. 2:**
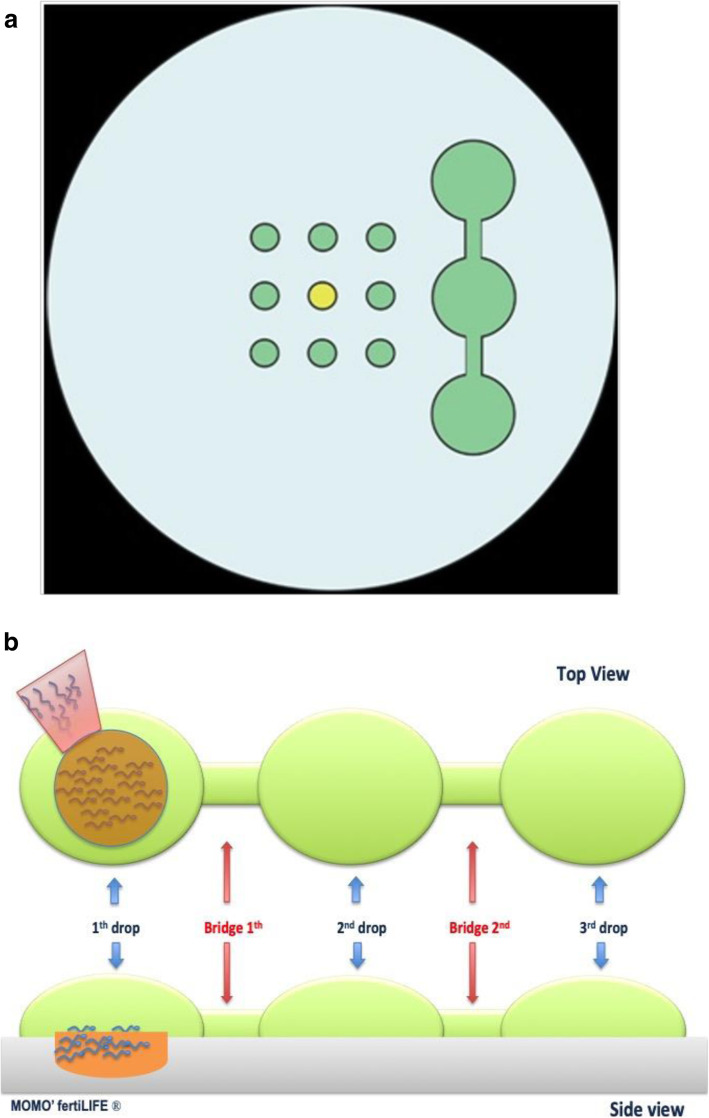
**a** ICSI plate scheme. At the centre are schematized culture medium containing oocytes drops (green colour) and PVP drop (yellow colour). On the right are depicted three additional drops linked by small culture medium in which the horizontal swim up is carried out. **b** Top and Side viewing of the sperm horizontal migration from the first drop (where the sperms are added) to the third drop (where the sperms are aspirated) trough 2 bridges that link them

Seminal fluids from Group B patients, were managed by the horizontal swim-up procedure. Briefly, the ICSI plate preparation included 3 additional 50 μl drops of G-mops® Vitrolife. The drops were linked through a small culture medium using a stripper pipette (Fig. 2a; b). In relation to the concentration and motility of the sperm sample, 1 to 5 μl of ejaculate were then injected in the proximal drop 10 min before presenting oocytes in separated drops (Table 2). During ICSI, adequate amounts of spermatozoa reached the superior edge of the distal drop, and some of them were subsequently recovered by needle and moved in a PVP drop and, subsequently, accurately selected for injection.

**Table 2:** Quantity of sample’s volume in μl to add in the horizontal migration in relation with concentration (N × 10^6^ sptz/ml) and motility (%A + B) of the sperm

*Concentration* *(N sptz/ml)*	*> 50% A + B*	*50–30% A + B*	*30–20% A + B*	*20–10% A + B*	*10–5% A + B*
≥ 20.000.000	1 μl	1 μl	1 μl	2 μl	3 μl
20.000.000–10.000.000	1 μl	1 μl	1 μl	2 μl	3 μl
10.000.000–5.000.000	2 μl	2 μl	2 μl	3 μl	3 μl
5.000.000–2.000.000	2 μl	2 μl	3 μl	3 μl	4 μl
2.000.000–1.000.000	3 μl	4 μl	4 μl	5 μl	5 μl

### Intra-cytoplasmic sperm injection (ICSI) and embryo transfer (ET)

In both groups, the ICSI procedure was performed at 37 °C under an inverted microscope (Nikon eclipse TE 200) at 400X magnification. The ICSI was completed by oil-hydraulic assisted microinjection system (Nikon eclipse TE 200). The ET procedure was performed after 3–5 days from OPU by using a catheter (Guardia Access Embryo Transfer Catheter, Cook Medical) with transabdominal ultrasound guidance (General Electric, Logiq V2) for both transfer and implantation of one to two embryos.

### Reproductive outcomes

Fertilization and cleavage rates were evaluated under an inverted microscope (Nikon eclipse TE 200). The presence of 2 pronuclei was assessed 16–18 h after ICSI and the embryo development was investigated 44–46 h after insemination. The cleavage was evaluated on day 3. Blood hCG evaluation was performed 12–14 days after ET to assess the pregnancy condition and women with positive test were monitored 2 weeks later by transvaginal ultrasound examination. Ongoing pregnancies were revealed by the presence of gestational sac and fetal viability at the week 4th after transfer.

### Data analysis and statistics

The randomization frequency was generated by the statistic software STATA 9.0, whereas the allocation sequences were kept hidden to both researchers and the staff in charge of the statistical analysis of samples. A gynaecologist doctor not involved in the treatments, recruited the participants following the mentioned randomization criteria. The study was ‘double-blinded’ for both couples and researchers. To obtain an accurate statistical assessment of the samples, general characteristics, geographic regions and clinic data such as age, geographic area, ethnicity, reasons and length of infertility, were punctually analysed (Table 1). Mean values of MII oocyte number, oocyte inseminated number and the initial total sperms counts of the two groups were compared (Table 3). Furthermore, data from both groups were evaluated and matched in terms of timing procedures fertilization, cleavage, blastocyst, implantation, clinical pregnancy and ongoing pregnancy rates.

**Table 3:** Patient’s characteristics and in reproductive outcomes of the two groups included in this study

	A Group (***n*** = 498)	B Group (***n*** = 536)	***P*** value
Basal sperm concentration (×1 0[5]/ml)	34.9 (±24.65)	36.01 (±25.81)	0.67
Retrieved MII oocytes (number)	5.44 (±2.67)	5.6 (±3.29)	0.63
Injected MII oocytes (number)	5.21 (±2.46)	5.45 (±3.03)	0.42
Timing procedures **(**minutes)	61 (±12)	12 (±6)	0,0001
Fertilization rate	80.00% (±18.35)	78.87% (±18.87)	0.56
**Cleavage rate**	92.10% (±13.69)	98.22% (±8.58)	0.0003
**Blastocyst rate**	41.2% (±20.69)	48.1% (±18.79)	0.0010
Implantation rate	20.25% (±30.90)	24.57% (±32.52)	0.19
Clinical pregnancy/cycle (%)	164/498 (32.8%)	209/536 (38.9%)	0.22
Ongoing pregnancy/cycle (%)	131/498 (26.4%)	172/536 (32.1%)	0.23

Note: Value are expressed as mean ± sd or percentage

Therefore, data were expressed as mean ± standard deviation (M ± SD) for continuous variables, while percentages were used for categorical variables (Table 3). Mean values were compared by Student’s t test. Percentages were compared by Chi-squared test. *P* value < 0.05 was considered significant using both statistical analyses. Data analysis was performed by using Statistica version 8.0 (StatSoft Italia Srl, Padova).

## Results

We completed 1034 ICSI cycles with 1898 embryos transferred. ETs were performed in day 3 for both Groups (total number 413) and day 5 (total number 621). In particular, 195 ETs at day 3 and 298 at day 5 were completed in Group A, whereas 218 ETs at day 3 and 323 at day 5, in Group B.

**Fig. 3:**
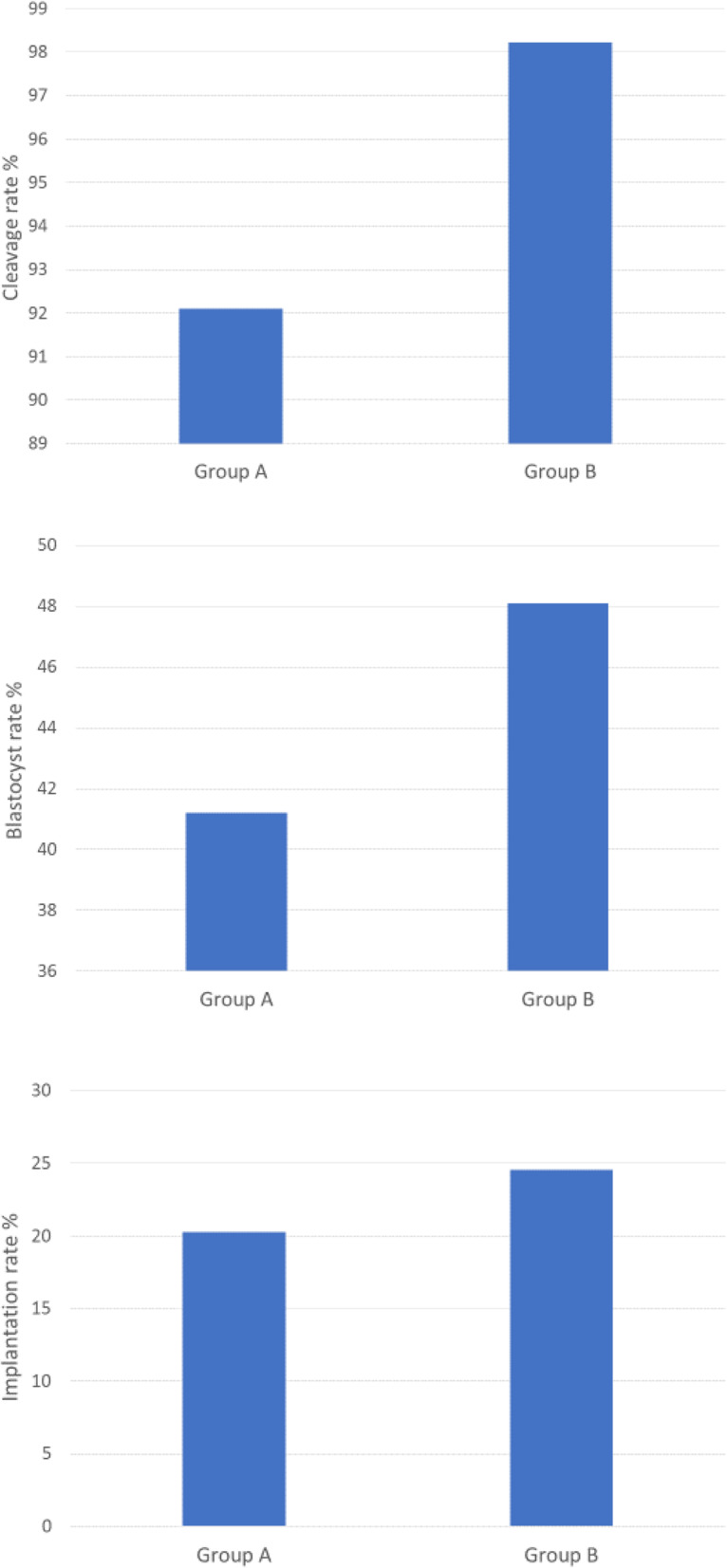
Bar chart 1 Cleavage rate % in Group A and Group B; Bar chart 2: Blastocyst rate% in Group A and group B; Bar chart 3: Implantation rate % in Group A and Group B

**Fig. 4:**
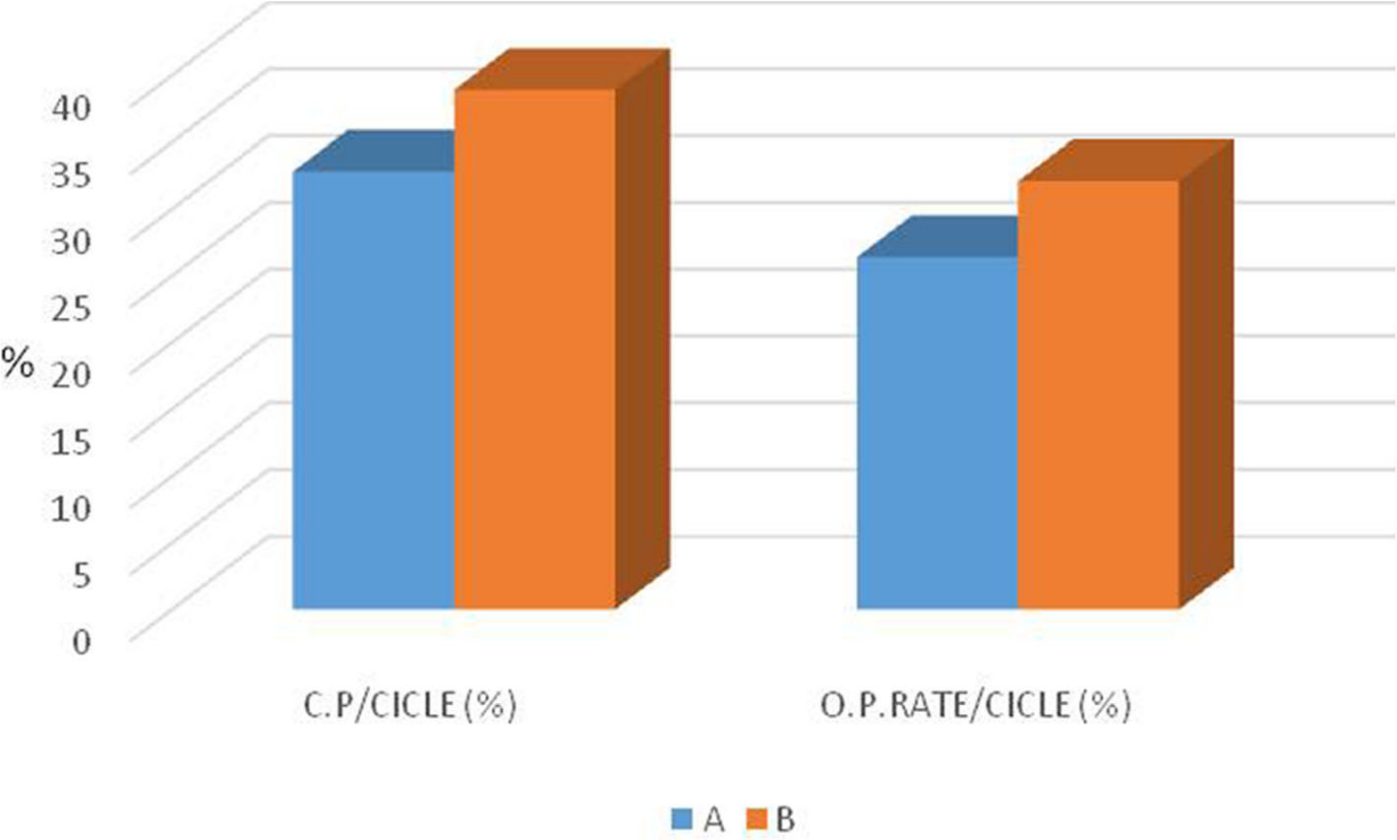
Bar Chart: Clinical Pregnancy Rate and Ongoing Pregnancy Rate in group A and group B

As shown in Table 3, there were no significant differences between the two groups in terms of MII oocyte number, oocyte inseminated number and initially detected sperm concentrations in millions. By contrast, the timing procedures were significantly different between both groups with a relevant time-saving in group B compared to group A (Table 3). Mean values of fertilization, cleavage, blastocyst formation and implantation rates from each group are listed in Table 3 and represented in Fig. 3 (Bar chart 1,2,3). No statistical differences for fertilization rate and implantation rate (*P* > 0.05) were found, whereas both cleavage and blastocyst rates were significantly higher in the group using the novel sperm preparation technique (B). Finally, the clinical pregnancy as well as the ongoing pregnancy rates did not show any statistical differences between the two groups. The results are represented in Table 3 and shown in Fig. 4.

## Discussion

Immediately after ejaculation, sperm is unable to complete fertilization due to the occurrence of immature and necrotic sperm cells as well as blood cells and seminal fluid in the sample, which produces bioactive or toxic endogenous substances. In addition, the collected sperms are exposed to exogenous factors acting as potential sources of ROS, including the effects of visible light, pH and temperature, as well as centrifugation during the spermatozoa preparation [22].

Therefore, an ideal sperm preparation technique aims to select highly motile, morphologically normal spermatozoa with a minimal DNA damage in a fast, safe and cost-effective manner. Despite several procedures of semen preparation for ART have been developed [17], the well-adopted swim-up technique is unable to provide sperms without DNA damages. In fact, a significant decrease in percentage of normally chromatin-condensed spermatozoa has been reported as recurrent following this procedure [23].

Here, we report a simpler and faster procedure for semen preparation based on horizontal sperm migration directly in the injection dish. We compared the ICSI-ET outcomes of this new sperm arrangement with the conventional swim-up technique and found that between the two methods there were no statistical differences in fertilization and implantation rate. Surprisingly, cleavage and blastocyst rates were significantly higher in the group treated with the new method thus implying lower level of fragmented DNA in sperms in accordance with some studies describing that a low DNA Fragmentation Index (DFI) correlates with an increased cleavage rate (Jiang [7, 24]). However, DFI was not investigated in our study. Moreover, it has been reported that embryos obtained from spermatozoa which are exposed to a minor or minimal centrifugation-stress, could have an improved blastocyst development as effect of the major paternal contribution to the development of a normal embryo genome [25]. In line with this observation, we found that both clinical and ongoing pregnancy rates were numerically higher in couples who underwent our innovative approach (group B) although no significant differences were recorded with respect to the group treated with swim-up method (A).

The time saving of the investigated novel technique which is much faster than the classical sperm preparation procedure, is relevant particularly in avoiding the centrifugation steps using density gradients usually requiring up to 20 min, as well as subsequent centrifugations with the culture media to wash the pellet and remove any gradient compound that means more than 10 min, and finally the swim-up from pellet which takes normally between 40 and 50 min [26, 27]. On the contrary, the novel procedure here reported, requires a total of 10 min to prepare the sperm, with a time saving of about 50 min compared with the direct swim-up procedure and further 75–80 min when including the density gradient step in the preparation. On the other hand, this procedure is cheaper for avoiding the cost of media that need to be necessarily used with the classical technique of sperm preparation while it has also been demonstrated in the absence of bacterial contaminations [28, 29].

Furthermore, the lower rate of mismatching error between different semen samples of this innovative procedure, should be considered a remarkable aspect to improve processes related to patient identification. However, although in absence of statistically relevant differences, data from direct horizontal sperm migration in injection dishes in terms of pregnancy rate seem to be equal or even better if compared to the conventional procedure while confirming its effectiveness in terms of ICSI outcomes without reducing the fertilization rate [30, 31].

Finally, we would like to emphasize that the statistical assessment was completed in equally distributed groups while a putative weakness of the study is related to the embryo quality, rating as results from data related to the ETs at day 3 and day 5 from both groups. However, data related to both implantation and pregnancy rates of at day 3 and day 5 are not shown since no statistical differences were found.

## Conclusion

In conclusion the horizontal sperm migration directly in the injection dish leads to considerable advantages as it is time-sparing, economical and does not need the involvement of the employed laboratory staff dedicated to the sperm preparation, while allowing to fruitfully work with lower concentration of motile sperms. Based on our favourable data, we plan to apply this method to more patients and to comparatively explore the role of ROS in different techniques of sperm preparations.

## Data Availability

The datasets analysed during the current study are available from the corresponding author on reasonable request.
